# In-Vitro Identification and In-Vivo Confirmation of DNA Methylation Biomarkers for Urothelial Cancer

**DOI:** 10.3390/biomedicines8080233

**Published:** 2020-07-22

**Authors:** Christina U. Köhler, Michael Walter, Kerstin Lang, Sabine Plöttner, Florian Roghmann, Joachim Noldus, Andrea Tannapfel, Yu Chun Tam, Heiko U. Käfferlein, Thomas Brüning

**Affiliations:** 1 Institute for Prevention and Occupational Medicine of the German Social Accident Insurance, Institute of the Ruhr University Bochum (IPA), Bürkle-de-la-Camp Platz 1, 44789 Bochum, Germany; koehler@ipa-dguv.de (C.U.K.); lang@ipa-dguv.de (K.L.); ploettner@ipa-dguv.de (S.P.); bruening@ipa-dguv.de (T.B.); 2 C.ATG Core Facility for NGS and Microarrays, University of Tübingen, Calwerstr. 7, 72076 Tübingen, Germany; michael.walter@agilent.com; 3 Department of Urology, Marien Hospital Herne, University Hospital of the Ruhr University Bochum, Hölkeskampring 40, 44625 Herne, Germany; florian.roghmann@elisabethgruppe.de (F.R.); joachim.noldus@elisabethgruppe.de (J.N.); 4 Institute of Pathology, Georgius Agricola Foundation Ruhr, Ruhr University Bochum, Bürkle-de-la-Camp Platz 1, 44789 Bochum, Germany; andrea.tannapfel@rub.de (A.T.); yu.tam@rub.de (Y.C.T.)

**Keywords:** DNA methylation, urothelial cancer, biomarkers, cell lines, primary cells, urine

## Abstract

We identified DNA methylation targets specific for urothelial cancer (UC) by genome-wide methylation difference analysis of human urothelial (RT4, J82, 5637), prostate (LNCAP, DU-145, PC3) and renal (RCC-KP, CAKI-2, CAL-54) cancer cell lines with their respective primary epithelial cells. A large overlap of differentially methylated targets between all organs was observed and 40 Cytosine-phosphate-Guanine motifs (CpGs) were only specific for UC cells. Of those sites, two also showed high methylation differences (≥47%) in vivo when we further compared our data to those previously obtained in our array-based analyses of urine samples in 12 UC patients and 12 controls. Using mass spectrometry, we finally assessed seven CpG sites in this “bladder-specific” region of interest in urine samples of patients with urothelial (*n* = 293), prostate (*n* = 75) and renal (*n* = 23) cancer, and 143 controls. DNA methylation was significantly increased in UC compared to non-UC individuals. The differences were more pronounced for males rather than females. Male UC cases could be distinguished from non-UC individuals with >30% sensitivity at 95% specificity (Area under the curve (AUC) 0.85). In summary, methylation sites highly specific in UC cell lines were also specific in urine samples of UC patients showing that in-vitro data can be successfully used to identify biomarker candidates of in-vivo relevance.

## 1. Introduction

Urothelial cancer (UC) is the most frequent neoplasm of the urothelial tract. Advanced age, smoking, male gender, and exposure to certain chemicals at the workplace or via the environment such as aromatic amines or arsenic are well-accepted risk factors [1]. The majority of tumors present superficially and can be well excised by transurethral resection. However, recurrence rates of up to 70% require timely follow-up examinations to prevent rare cases of progression, virtually making UC a chronic disease [2].

Current guidelines suggest invasive urethrocystoscopy accompanied by cytology as the standard of care in symptomatic persons for diagnosing de-novo and recurrent UC and independent on stage, grade or risk of recurrence. However, many patients, particularly men, experience significant discomfort, secondary hematuria, and infections associated with de-novo or recurrent UC diagnosis due to invasive cystoscopy [3,4]. To minimize the number of cystoscopies, numerous approaches to non-invasively diagnose UC have been developed in urine. Genetic approaches such as analyzing DNA mutations and DNA methylation signatures, among others, have been shown to be most promising [5,6,7].

Currently, molecular markers are not part of clinical guidelines, as appropriate studies showing equivalence with cystoscopy are lacking. However, as it was shown that the knowledge of a positive test result significantly increases the number of detected bladder tumors, they might be useful as adjunct tests ideally performed prior to cystoscopy [8]. In addition, they might be useful to detect UC when used in the surveillance of high risk individuals based on male gender, advanced age, high pack years of smoking, occupational exposures to bladder carcinogens or a combination thereof.

Although the refinement of molecular technologies enables the analysis of cell-free DNA from urine, molecular approaches employing DNA from the urinary cell pellet display similar or even superior diagnostic value due to a substantial shedding of tumor cells into the urine [9,10]. However, urine does not only accumulate urothelial cells, but also cells from the prostate and kidney [5,11], making it necessary to account for potential cross-specificities of biomarkers. Because neoplasia of different organs including urological cancers also often share common molecular features [12], the urinary accumulation of cells stemming from different urological tissues has previously been utilized to identify biomarkers that, overall, detect urothelial, prostate and renal cancer [13]. Conversely, it remains challenging to find cancer biomarkers that are specific for a particular single organ or tissue such as the urothelium [14]. Avoiding cross-specificities is of special importance in men who have an increased risk for multiple urological malignancies (in particular bladder and prostate).

In the present study, we aimed to identify urinary DNA methylation biomarkers that are capable of distinguishing cancerous urothelial cells from those of the prostate and kidney. For this purpose, we applied a genome-wide, array-based screening to various urological tumor cell lines of urothelial, prostate and renal origin and compared the results to those obtained from the respective non-tumorous primary cells. To select targets of actual relevance for clinical UC detection, we further compared our in vitro results to those previously obtained in vivo from a genome-wide screening of a small set of urine samples from UC patients and controls [15]. Finally, candidate CpG-sites were evaluated in vivo in a larger set of urine specimens from UC patients, patients with prostate (PC) and renal cancer (RC), and non-cancer controls (population (PCt) and urological hospital controls (UCt)).

## 2. Materials and Methods

### 2.1. Cell Lines and Cell Culture

Three cancer cell lines and one primary cell culture (all male donors) were used of each of the following tissues: bladder, prostate and kidney (Table 1). The cell lines represented different individualities of the respective tissues in terms of grading, staging, metastatic potential, tumor shape and cellular origin. Due to the increased risk of UC in elderly men we chose cell lines and primary cells accordingly where possible.

The cell culture was performed according to the recommendations of the respective supplier. Culture media for the different cancer cell lines were obtained from PAN-Biotech (Aidenbach, Germany) and supplemented with varying concentrations of heat inactivated fetal calf serum (FCS; Biochrom, Berlin, Germany) as stated in Table 1. All media for the cultivation of cell lines contained 1% penicillin/streptomycin (PAN-Biotech, P06-07100).

For CAL-54 cell line culture, hydrocortisone and epidermal growth factor (EGF) was purchased from AppliChem GmbH (Darmstadt, Germany) and Hölzel Diagnostika Handels GmbH (Köln, Germany), respectively. For the primary cells, special kits of the respective providers were used (Table 1). Both primary cells and cell lines were taken into culture directly after delivery from the provider and cultivated under standard conditions (5% CO_2_, 37 °C). After a few passages (2–4 for primary cells and less than 10 for cell lines) cells were harvested, centrifuged at 200× *g* for 5 min, re-suspended in Phosphate-buffered saline (PBS) and stored at −80 °C until used for DNA-extraction and methylation analyses.

### 2.2. DNA Isolation from Cell Pellets

DNA was isolated from about 1 to 5 million cells reconstituted in 200 µL PBS using the QIAamp DNA Mini Kit (Qiagen, Hilden, Germany, Cat.-No. 51304) and following the recommendations of the manufacturer. DNA was eluted in 50–200 µL PBS and stored at −20 °C until further use.

### 2.3. DNA Methylation Arrays in Cell Culture

The genome-wide DNA methylation landscape of all cells was determined by Infinium Methylation 450 K arrays (Illumina, San Diego, CA, USA). For this purpose, 700 ng DNA were bisulfite converted using the EZ-DNA methylation kit as recommended by the manufacturer (Zymo Research, Irvine, CA, USA).

DNA was subject to whole genome amplification and enzymatically fragmented according to the HD Methylation Assay Protocol Guide (15019519 B). Then, the methylation status of >450,000 CpG sites was assessed by means of allele-specific primer annealing and subsequent single-base extension with fluorescence labeled nucleotides. All reactions were performed according to the recommendations of the array-manufacturer. For each interrogated DNA-site, the fluorescence intensities of replicate beads were averaged using GenomeStudio V2011.1. Quality control and statistical evaluation was conducted by the use of R-2.14.0 and various Bioconductor packages (v.2.16.0) [16]. Array results were preprocessed using the Bioconductor package “lumi”. Channel intensities were color adjusted and the resulting data were normalized using the option ‘simple scaling’ [17]. The intensities of methylated and non-methylated probes at each individual CpG were used to calculate beta values that represent the methylation level of the respective locus. As average beta values (not normally distributed) are not suitable for statistical analysis, the log ratios of the intensities of methylated C’s over non-methylated C’s (M-values) were calculated [18]. The raw data (i.e., non-normalized intensities) of both screening arrays are available from the Gene Expression Omnibus GEO functional genomics public depository (Accession GSE149387).

### 2.4. Evaluation of the Cell Culture Array Results

To determine targets that were differentially methylated in urothelial, prostate or renal cancer cell lines when compared to the respective primary cells, a linear model was developed using the Bioconductor package‚ limma’ [19]. Based on the M values, we calculated the coefficients of the linear model that describe the methylation profile of the respective locus. The relevant comparisons were defined as a contrast matrix and an F-statistic was calculated for the comparison of cancer cell lines and the respective primary cells. Standard errors were moderated by an empiric Bayesian model [20]. Subsequently, *p*-values from the F-statistics were calculated and corrected for multiple testing using Benjamini-Hochberg multiple testing correction. A decision matrix was created to identify sites of significant differential methylation in the individual comparisons. In addition, the beta values from the three cell lines were averaged and subtracted from the beta values of the primary cells to characterize the average difference between cancer and non-malignant cells. All targets with a corrected *p*-value < 0.05 were selected for further evaluation.

To select targets that were differentially regulated specifically in UC cell lines, the targets displaying methylation differences between UC cell lines and primary urothelial cells were further investigated. Therefore, a second linear model was established where the bladder cancer cell lines were compared to a group comprising of all renal and prostate derived cell lines and all primary cells. *p*-values were corrected for multiple testing as above and targets with *p*-values < 0.05 were considered significant.

### 2.5. Comparison of In Vitro and In Vivo Array Data

To check whether potentially bladder-specific sites identified in cell culture arrays were also differentially regulated between urine specimens from UC patients and controls and thus potentially suitable for the clinical practice, we compared the results from the cell culture screening with our previously published in vivo array results of a differential analysis derived from 12 urine samples from UC patients and 12 age, gender and smoking-status matched non-UC controls [15], GEO public depository: Accession GSE 120288).

### 2.6. In Vivo Confirmation in Additional Urine Specimens

The two potentially bladder-specific CpGs which were identified by the in-vitro/in-vivo comparison were analyzed together with five neighboring CpGs by quantitative mass spectrometry in urine from 293 UC patients (232 male, 61 female), 75 PC patients, 23 RC patients (14 male, 9 female), 44 population controls (33 male, 11 female) and 99 urological controls (70 male, 29 female) (Table S1). The latter consisted of patients that underwent transurethral resection due to initial suspicion of UC and suspicious-appearing tissue sections in the bladder during transurethral resection. However, the histological examination of the tissue samples ultimately did not confirm the initial suspicion. As previously outlined [21], albeit being representative, our UC patient population belongs to a low risk collective and, consequently, contains a higher number of patients with low-grade pT1 tumors (Table S1).

All analyses of human materials were approved by the Ethics Committee of the Ruhr-University Bochum (No. 3674-10 and No. 4785-13). The study followed the Declaration of Helsinki and all participants provided written informed consent.

Final statistical evaluation was carried out in a subset of the aforementioned collective due to our previously published observations that a strong contamination with leukocytes suppresses UC-dependent DNA methylation signals and UC history (de-novo, recurrent) is also an important influencing factor of DNA methylation in cancer cases [15]. Consequently, urine specimens with ≥500 leukocytes/µL and those cancer cases with lacking information on UC history were excluded. Overall, the final collective to evaluate the diagnostic potential of the amplicon comprised 249 UC patients (207 male, 42 female), 71 PC patients, 21 RC patients (13 male, 8 female), 41 population controls (30 male, 11 female), and 68 urological controls (52 male, 16 female) (Table S2).

### 2.7. Preparation of DNA from Urine

The preparation of DNA has been described in our previous study in detail [15]. In brief, the urinary sediment was prepared by centrifugation of the voided urine, washing and resolving the pellet with PBS. DNA was isolated using the QIAmp MinElute Virus Spin Kit (Qiagen, Hilden, Germany). Digestion of RNA was performed by incubating the samples with DNase-free RNase (Roche, Mannheim, Germany). DNA was purified by the Clean and Concentrator TM-25 Kit (Zymo Research, Irvine, CA, USA), eluted using TE buffer (AppliChem, Darmstadt, Germany), and stored at −20 °C until further analysis.

### 2.8. Quantitative Mass Spectrometry of DNA Methylation

The potentially bladder specific target region deduced from the comparison of the screening experiments in cell culture and urine was assessed in more detail by matrix-assisted laser desorption/ionization–time-of-flight (MALDI-TOF) mass spectrometry (MassARRAY EpiTYPER system, Agena Bioscience GmbH, Hamburg, Germany) which enables the quantitative measurement of CpG methylation at single dinucleotide resolution [22,23]. For this purpose, primers covering a DNA-stretch which includes the two most promising candidate sites were designed by Agena`s EpiDesigner software (http://www.epidesigner.com/index.html). The sequences were aggaagagagGGGTTATGTTGAGAAGTAAGGAATGT (forward-primer) and cagtaatacgactcactatagggagaaggctCCCACACAAAACTTAAAAATAAAACTT (reverse primer, the small print represents the respective tags required by the method). The amplified region was CHR6:28911328-28911620 (genome build GRCh37/hg19). All analyses were carried out according to the protocol of Agena Bioscience GmbH and have been previously described in detail [15].

### 2.9. Statistics and Modeling

Differences in DNA methylation levels between groups were calculated using analysis of variance (ANOVA, Kruskal–Wallis Test) for group comparisons and the Mann–Whitney Test for individual comparisons. *p*-values < 0.05 were considered statistically significant. To determine the diagnostic performance of each CpG, Receiver Operating Characteristic (ROC) curves were calculated and the areas under the curves (AUCs) were determined. In addition, the sensitivities for the detection of UC were determined at a pre-set high specificity (95%), because specificity is considered important when developing diagnostic biomarkers to avoid false-positive results. All calculations were performed by using Graph Pad Prism software, version 8.1.0 (Graph Pad Software, San Diego, CA, USA).

## 3. Results and Discussion

### 3.1. Cell Line Screening Reveals Large Overlap of Differentially Methylated Sites between Cancer Types

**Figure 1:**
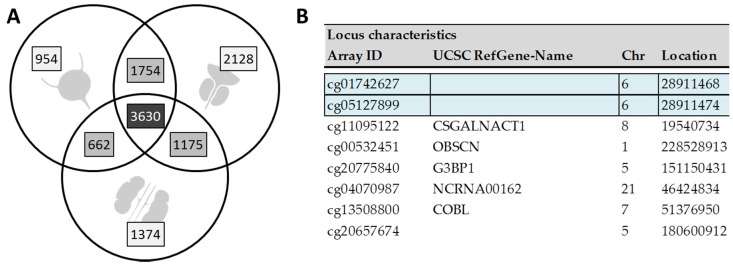
(**A**) Differential methylation analysis between urothelial (upper left), prostate (upper right) and renal (lower middle) cancer cells and their respective primary cells and overlap of the results. Overall, methylation differences at 4,676,979 target sites were evaluated. (**B**) Locus characteristics of the eight target sites which were urothelial cancer (UC)-specific in both, the in vitro and the in vivo evaluations. The top two (intergenic) sites were chosen for additional in-vivo confirmation by mass spectrometry.

An initial screening approach comparing urothelial, prostate, and renal cancer cell lines with corresponding primary cells from unrelated individuals revealed 7000, 8687, and 6841 target sites which were differentially methylated between malignant and non-malignant cells of the respective tissues. The strongest overlap in regulated targets was observed between urothelial and prostate cancer cells (5384 sites) followed by prostate and renal (4805) and urothelial and renal cancer cells (4292). The overall overlap between targets in all three organs (3630) was also still large (Figure 1A). This finding is in line with previous tissue-based studies observing high degrees of congruence between different kinds of cancers, proving remarkable similarities in the affected pathways between neoplasms of distinct tissues [12,24].

Among the 7000 sites differentially methylated between non-cancerous and cancerous urothelial cells, the vast majority (94%, 6577 sites) were hypermethylated in UC. Of those, 84% (5525 sites) displayed a methylation difference of >20%; while 56% (3683 sites) showed a methylation difference of at least 40%. Among the 423 hypomethylated sites, 66% (279 sites) showed strong (>40%) and 93% (393 sites) at least moderate (>20%) differences in DNA methylation.

### 3.2. Few Targets Are Specific for Urothelial Cancer Cell Lines Only

Methylation differences of 40 targets were specific for urothelial cancer cells only in terms of equally well discriminating UC cell lines from PC and RC cell lines and all primary cells (Table S3). Thirty-one sites were hypermethylated in UC with 26 displaying methylation differences >40%. Of the nine hypomethylated sites, methylation differences exceeded 40% at seven sites.

### 3.3. Comparison with Previous In Vivo Data Identifies Eight Targets of Potential In Vivo Diagnostic Relevance

Regarding the 40 sites differentially methylated specifically in UC in vitro, we reviewed the results of our previously performed genome-wide arrays comparing the DNA-methylation in urine from UC-Patients with population controls (PCt) and urological controls (UCt) (Table S3) [15].

Eight out of the 40 sites in vitro also showed methylation differences of >20% in urine (Figure 1B, Table S3). Four of these sites, based on adjusted *p*-values, displayed statistically significant different urinary DNA methylation in the UC vs. PCt comparison (in the UC vs. UCt comparison, the adjusted *p*-value never fell below 0.05). Among these four sites, we selected cg01742627 (a genetic location where no protein-coding gene has been allocated) due to its highest observed difference (>50%) in DNA methylation between UC patients and any control group. Incidentally, the site with the second highest methylation difference (>47% in cg05127899) was located only six base-pairs upstream of cg01742627. Therefore, it was possible to assess and verify both targets within the same amplicon by mass spectrometry. As both CpG sites were located on the same fragment in the downstream RNA-cleavage, an integrated methylation result was obtained for both CpGs (CpG unit 5.6). Mass spectrometry of the respective amplicon also yielded unambiguous mass results for five additional and neighboring CpGs (CpGs 1, 7, 8, 12 and 13) in this region of interest. However, CpGs 1 and 13 were excluded from all following statistical evaluations due to a high number of missings in the MS data. Overall, we designated the identified region “BLSP” for its high likely-hood of being “bladder specific”.

### 3.4. DNA Methylation Is Significantly Increased in Urine of UC Patients

**Figure 2:**
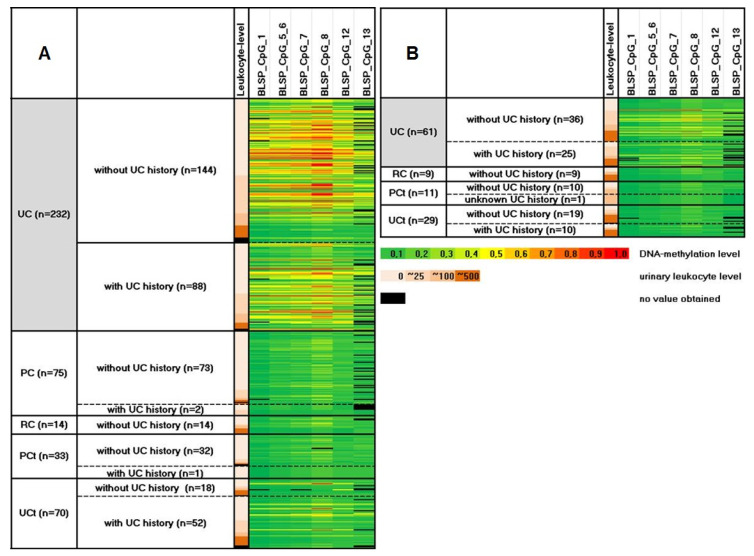
Cytosine-phosphate-Guanine motifs (CpG)-resolved DNA methylation for the potentially bladder specific (BLSP) amplicon in urine from 424 male (**A**) and 110 female (**B**) patients with urothelial (UC), prostate (PC) and renal cancer (RC) and population (PCt) and urological controls (UCt). Increasing DNA methylation values are encoded by a color gradient from 0% methylated (green) to 100% methylated (red). The collectives are stratified for UC history (de-novo and recurrent UC) and urinary leukocyte counts (increasing leukocyte counts from top to bottom in each group as indicated by increasing orange shades). CpGs 1 and 13 are shown here, but excluded from all following statistical evaluations due to the higher number of missings (where no values have been obtained).

According to the results from our screening in cell culture, we studied the methylation differences of the BLSP amplicon between all groups in all available urine samples from UC patients and compared the results to those of PC and RC patients as well as controls with non-malignant urological diseases (UCt) and population controls (PCt) (Tables S1 and S4, Figure 2).

We observed a notable and significant hypermethylation of the BLSP amplicon in the urinary sediment from UC patients when compared to urinary cells from PC and RC patients. Like in our previous study [15], the differences were most pronounced for male UC patients (median differences 21% and 20%, median *p*-values < 0.0001 and 0.0003 across all CpGs) rather than females. In addition, the BLSP amplicon was also strongly hypermethylated in the urinary sediment from male UC patients when compared to PCt (median difference 20%, median *p*-value < 0.0001 across all CpGs) and UCt (median difference 23%, median *p*-value < 0.0001 across CpGs). Again, the differences between female UC patients and non-cancer controls were less pronounced (median differences 5% and 3%, median *p*-values 0.0025 and 0.1503 across all CpGs for the UC vs. UCt and UC vs. PCt comparison). As outlined previously [15], we assume that these reduced differences are gender-related due to a dilution of methylated DNA of UC-origin in females by non-methylated DNA from leukocytes or squamous epithelial cells that occurs more frequently in urine of women. In accordance with these results, the differences in DNA methylation levels between female UC and RC patients were also less pronounced (median difference 2% and median *p*-value 0.3142 across CpGs).

### 3.5. BLSP Methylation Is Largely Independent on Age, Stage and Grade in Healthy Controls and/or UC Patients

To study whether age has a substantial effect on DNA methylation of the BLSP amplicon in vivo at all (and independent on any disease) and whether or not the observed increased DNA methylation in urine of UC patients may be confounded by age, we first studied the influencing factor of age in all healthy controls (UCt, PCt). No significant correlation with age could be observed for any CpG in the BLSP amplicon, neither in men nor in women. In addition, no association with age could be observed in female UC patients. However, the BLSP amplicon displayed a small (median Spearman correlation coefficient 0.1934) but significant (median *p*-value 0.003) increase of DNA methylation in all CpGs of male UC patients with increasing age (Figure S1). Overall, these results suggest that the observed increase in DNA-methylation of UC patients is predominantly caused by the cancer (UC) rather than the advanced age of the patients.

Similarly to age, no differences in methylation levels between high or low grade UC and no increases of DNA methylation with stage could be observed in UC patients, neither in men nor in women (Figure S2). This result was to be expected based on our identification strategy in vitro which involved cell lines covering a broad tumor spectrum. Nevertheless, the DNA methylation of T1 patients in urine was consistently higher in the BLSP amplicon than the methylation in urine of Ta and T2 patients.

### 3.6. BLSP Methylation Is also Influenced by UC History and by Leukocyte Counts in Male UC Patients

**Figure 3:**
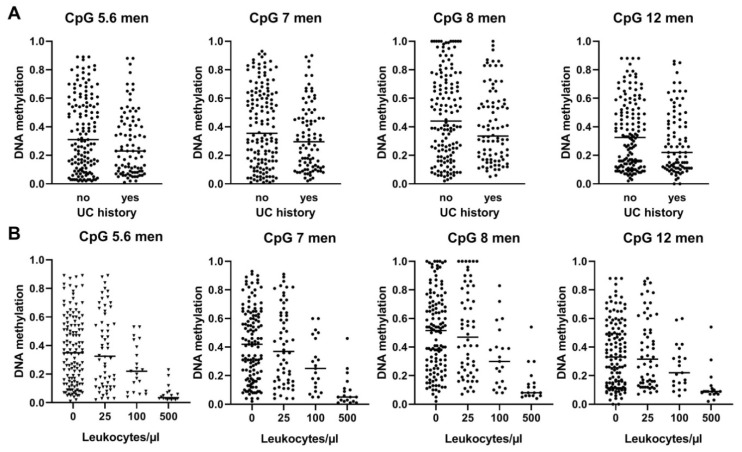
Urinary DNA methylation values of male UC patients from the large urine dataset (Table S1); the data are stratified for UC history (**A**) and urinary leukocyte counts (**B**). Horizontal bars represent the median DNA methylation value.

The median DNA methylation across all CpGs was, albeit not significantly, slightly and consistently lower in urine of male UC patients with recurrent UC when compared to primary UC (median *p*-value 0.08935, median 9.25% lower DNA methylation across CpGs) (Figure 2 and Figure 3A). The weaker performance of cell-based markers in urine specimens from patients with recurrent UC is a well-described phenomenon and is most likely attributed to the fact that recurrent tumors are smaller in size and release less cells into the urine [25]. More importantly, DNA methylation was strongly reduced in urine samples from male donors with high leukocyte counts, i.e., median 34.3% lower DNA methylation in samples with >500 leukocytes/µL across all CpGs (*p* < 0.0001, Figure 2 and Figure 3B). This finding confirms our own previously reported results [15] and those of other research groups that also reported diminished urinary biomarker signals in leukocyte-rich urines [9].

As only <8% (18 out of 232) of the urine specimens in male UC patients contained >500 leukocytes/µL urine (Table S1), we assume that only a minor share of urine specimens would have to be excluded from BLSP analysis in clinical practice due to their high leukocyte concentration. However, a second specimen (taken shortly after the first sample because the leukocyte count can be quickly checked by dip stick analyses on-site) still might enable a conclusive analysis for the respective patient.

Interestingly, an impact of UC history and leukocyte count on the DNA methylation level could not be observed in urine from women, most likely due to the concealing effect of other cellular urine components like squamous urothelial cells which has been discussed above (Figure S3). One option to overcome the disturbing effect of leukocytes on the marker performance appears to be the depletion of the urinary specimen from contaminating white blood cells. However, a separation of cells requires the processing of fresh urine which is not possible in the majority of clinical settings and would thus negatively affect our marker’s applicability. Instead, we believe that a marker must tolerate robust pre-analytical handling until the sample reaches the site of analysis. The latter can be fulfilled by DNA-methylation analysis from freshly obtained urine or urinary cell pellet, even if the material was frozen intermediately.

### 3.7. The Methylation of the BLSP Amplicon in Urine Is of Diagnostic Relevance in Men

Due to the significant relevance of extremely high urinary leukocyte levels and UC history, we decided to evaluate the diagnostic potential of the BLSP amplicon in a reduced dataset excluding specimens >500 leukocytes/µL and those where information on UC history was missing. (Table S2).

**Figure 4:**
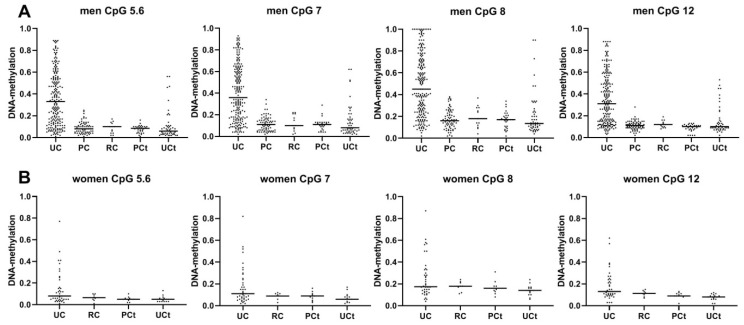
Urinary DNA methylation values of urine from male (**A**) and female (**B**) study participants; comparison of all groups (UC, PC, RC, UCt, PCt) in the reduced dataset (excluding urines with >500 leukocytes/µL); horizontal bars represent the median DNA methylation value.

Similar to what was observed for the complete sample set, DNA methylation within the BLSP amplicon was significantly higher in male rather than female UC patients. Thus, a significant UC-specific hypermethylation could be observed in men only when comparing UC to PC and RC patients (median *p* < 0.0001 and median difference 25% across CpGs for both comparisons), and to PCt and UCt controls (median *p* < 0.0001 for both comparisons and median differences of 25% for the UC vs. PCt and 28% for the UC vs. UCt comparison and across CpGs) (Figure 4). The strongest differences were observed for CpG 8 (Table S5).

**Figure 5:**
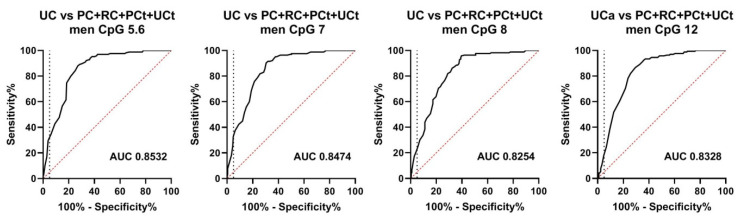
Receiver operator characteristic (ROC) curves for the diagnosis of UC patients in the reduced urine dataset among non-UC control individuals consisting of prostate (PC) and renal cancer (RC) patients and population (PCt) and urological controls (UCt). The vertical dashed lines indicate 95% specificity.

As an ANOVA-analysis did not reveal significant differences among the non-UC groups (i.e., PC, RC, PCt, and UCt) (Figure 4, Table S6), we combined all of these sample sets into one “non-UC” group (∑other groups). In order to evaluate the diagnostic value of BLSP in terms of tissue-specificity, we compared this integrated group to all UC cases and observed a highly significant (*p* < 0.0001) difference for all CpGs (Table S5). The median difference was 26% across all CpGs. The resulting ROC curves displayed similar AUCs (0.83–0.85) for all analysed CpGs. The highest AUC was observed for CpG unit 5.6 (Figure 5). At a pre-set specificity of 95%, the sensitivities for CpG unit 5.6 and CpG 7 were approximately 30% and 33%; whereas the sensitivities for CpGs 8 and 12 were approximately 21% and 18% (Table S7).

These sensitivities of the BLSP CpGs for the detection of UC at a pre-set 95% specificity were lower than those of previously identified CpGs in our in vivo study [15]. There, at a pre-set 95% specificity, the maximum sensitivities were 79% and 58% when comparing DNA methylation levels of UC patients to those of population and urological controls. Future studies should analyze whether a combination of different markers (including those within the BLSP amplicon) might increase their accuracy for diagnosing UC. Due to its tissue-specificity, a marker-combination involving BLSP might be of special interest for the surveillance of men who are at risk for multiple urological malignancies including UC and PC.

Interestingly, our previously identified UC-specific CpGs (in 10 amplicons) in vivo would have not been identified by the current cell-culture based screening approach. When assessing the cancer cell lines and the primary cells, the results for these CpGs show that at least one of the prostate or renal cell lines shares similar methylation-patterns with urothelial cell lines (Figure S4) and thus would not have been selected for further evaluation. Therefore, our in-vitro/in-vivo comparison presented here complements our previously published in vivo data by adding another UC-associated region of interest to our diagnostic spectrum of UC-specific DNA methylation sites.

### 3.8. The Methylation of the BLSP Amplicon in Urine Is of no Diagnostic Use in Women

**Figure 6:**
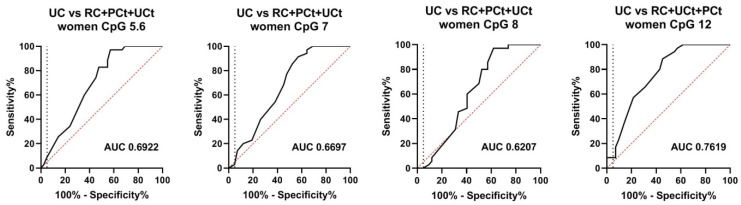
Receiver operator characteristic (ROC) curves for the identification of UC among females when compared to non-UC controls (RC, PCt and UCt). The vertical dashed lines indicate 95% specificity.

In contrast to men, DNA methylation across the BLSP-amplicon was very low in women including those with UC (Figure 4B). Therefore, the very small methylation differences (maximum 5%) between female UC patients and non-UC controls could not be interpreted as diagnostically meaningful, although, from a statistical point of view, significant *p*-values were observed for selected CpGs when comparing UC vs. PCt and UC vs. UCt (Table S5). Furthermore, only a small (1.5%, median) and not significant (median *p* = 0.3142) difference was observed when comparing female UC and RC patients (Table S5). Accordingly, the diagnostic performance of the CpGs in the BLSP amplicon is insufficient for their clinical use in female UC patients (AUCs 0.62–0.76, sensitivity below 9% at pre-set high specificities of ≥95%) (Figure 6). Additionally, the frequent presence of high leukocyte counts in urine specimens of postmenopausal women [26] (almost 30% (18 out of 61) in our collective, see Table S1) would disable far too many specimens from the analyses of the BLSP amplicon, rendering it not useful for its clinical application in women.

As already discussed above, we also ascribe the low methylation values observed across the BLSP amplicon in female urine to the presence of squamous epithelial cells in the female urine, similar to our previous observations in urinary DNA methylation levels of other amplicons in females [15]. Beyond this, we cannot exclude that differences in particular tumor characteristics between the male and female collectives might cause, at least in part, the reduced performance in women. However, conclusive analyses are limited by the small sample size due to the low incidence of UC in women.

### 3.9. Retrospective Analyses of Primary Urothelial Cells Reveal Potential Pre-Malignant and Age-Dependent Methylation Patterns

Interestingly, a retrospective evaluation of our previously identified methylation markers in vivo [15] also revealed sites of unexpectedly high levels of DNA methylation in the primary urothelial cells utilized in the present study such as in amplicons 2, 14, 42, and 64 (Figure S4). These findings are in line with the results of a combined analysis of the previous 450K array results in urine and those of the present 450K array cell culture data. The latter shows that primary urothelial cells preferably cluster with urine specimens of control subjects and those of renal and prostate primary cells, and that they also exhibit DNA methylation patterns which are similar to those of urine specimens from UC patients and from urothelial, prostate and renal cancer cell lines (Figure S5).

At the beginning of our study, we intentionally chose primary urothelial cells from male donors with advanced age due to the fact that UC occurs more frequently in elderly men. However, the above-mentioned finding, that the primary urothelial cells we used in our study already harbor certain characteristics of cells from UC patients and urogenital cancer cell lines, made us wonder whether methylation patterns within primary urothelial cells differs among donors of different age. Therefore, we also studied the DNA methylation of our previously identified in-vivo CpGs in primary urothelial cells of younger donors.

In concordance with previous studies reporting an increase in the level of DNA methylation or the number of methylated sites with age [27,28], a comparison of the utilized HBlEpC primary cells (donor age 69) with analogous primary cells of 14- and 30-year-old donors revealed an age-dependent increase in DNA methylation for the ten amplicons analyzed in our previous study (Figure S6). It is possible that our results for HBlEpC indicate premalignant lesions that were present at the molecular level but not histologically visible in the elderly cell culture donor. Our findings, once again, show the importance of using primary cells from elderly donors when screening for potential biomarkers in-vitro to minimize false positive identifications.

## 4. Conclusions

The newly identified bladder-specific DNA methylation biomarkers described here are capable of complementing hypermethylated DNA signatures of UC which have been previously identified in terms of combining them into a multimarker panel. Our study showed that appropriately designed in vitro screenings are suitable to identify hypermethylated DNA fragments that are specific for UC but not for other urological malignancies, making these targets suitable for later in-vivo use, e.g., in patients at risk of multiple urological malignancies in order to reveal the tumor-affected tissue.

## Figures and Tables

**Table 1:** Character of cell lines and primary cells, suppliers and culture conditions.

Cells	Supplier	Age ^1^	Origin and Characteristics [Medium, Additives and Kits]
RT4	CLS ^2^	63	**Bladder** transitional cell papilloma, G1-2, T1 [McCoy’s 5A, Pan-Biotec P04-06500, 10% FCS]
5637	DSMZ ^3^	68	Bladder transitional cell papilloma, G2 [RPMI 1640, Pan-Biotec P04-18500, 10% FCS]
J82	ATCC ^4^	58	Bladder transitional cell carcinoma, G3 T3 [MEM Eagle, Pan Biotech P04-09500, 10% FCS]
LNCAP	DSMZ ^3^	50	**Prostate** adenocarcinoma, lymph node metastasis, low metastatic potential [RPMI 1640, Pan Biotech P04-18500, 20% FCS]
DU-145	DSMZ ^3^	69	Prostate adenocarcinoma, brain metastasis, moderate metastatic potential [RPMI 1640, Pan Biotech P04-18500, 10% FCS]
PC-3	DSMZ ^3^	62	Prostate adenocarcinoma, bone metastasis, G4, high metastatic potential [Ham’s F12 Pan Biotech P04-15500/RPMI 1640, Pan Biotech P04-18500 1:1, 10% FCS]
RCC-KP	CLS ^2^	59	**Renal** clear-cell carcinoma, G3, pT3b, M1 [RPMI 1640 Pan Biotech P04-18500, 10% FCS]
CAKI-2	DSMZ ^3^	69	Renal papillary carcinoma [McCoy’s 5A, Pan Biotec P04-06500, 10% FCS]
CAL-54	DSMZ ^3^	75	Renal clear-cell carcinoma, metastatic pleura effusion [DMEM. Pan Biotech P04-01550, 20% FCS+, 0.04 µg/mL hydrocortisone + 10 ng/mL EGF]
HBIEpC	PeloBiotech ^5^	69	**Bladder**, primary epithelial cells [Epi growth medium, PeloBiotech PB 215-500, subculture Reagent Kit, PeloBiotech PB-090K]
HprEpC	PeloBiotech ^5^	69	**Prostate**, primary epithelial cells [Epi growth medium, PeloBiotech PB 215-500, subculture Reagent Kit, PeloBiotech PB-090K]
HREpC	PromoCell ^6^	77	**Renal**, primary epithelial cells [Renal epithelial cell growth medium 2, PromoCell C-26030, DetachKit C-41210]

^1^ Age of the donor; ^2^ CLS Cell Lines Services GmbH (Eppelheim, Germany); ^3^ German Collection of Microorganisms and Cell Cultures GmbH (Braunschweig, Germany); ^4^ American Type Culture Collection (Manassa, VA, USA); ^5^ PeloBiotech GmbH (Planegg, Germany); ^6^ PromoCell GmbH (Heidelberg, Germany).

## Supplementary Materials

Supplementary Materials can be found at www.mdpi.com/xxx/s1/.
