# Supplementary material for: In-Vitro Identification and In-Vivo Confirmation of DNA Methylation Biomarkers for Urothelial Cancer
Source: Biomedicines. 2020 Jul 22;8(8):233. doi: 10.3390/biomedicines8080233 (PMC7459535; doi:10.3390/biomedicines8080233)
Supplement: Supplementary file 1 [file biomedicines-08-00233-s001.pdf]

In-Vitro Identification and In-Vivo Confirmation of DNA Methylation Biomarkers for Urothelial Cancer

Christina U. Köhler, Michael Walter, Kerstin Lang, Sabine Plöttner, Florian Roghmann, Joachim Noldus, Andrea Tannapfel, Yu Chun Tam, Heiko U. Käfferlein and Thomas Brüning

Table S1. Patient and sample characteristics which are also the basis of Figure 2 in the main manuscript. The study population was stratified for gender, history of urothelial carcinoma, tumour classification and urinary leukocyte counts. UC=urothelial carcinoma; UCt=urological controls; PCt=population controls; PC=prostate cancer; RC=renal cancer; Cis=carcinoma in situ.

	**UC**		**PC**	**RC**		**PCt**		**UCt**	
	**male**	**female**	**male**	**male**	**female**	**male**	**female**	**male**	**female**
n	232	61	75	14	9	33	11	70	29
Age in years; median (range)	73 (35-93)	73 (48-92)	68 (48-82)	71 (50-83)	68 (59-82)	70 (48-87)	66 (46-80)	72 (45-92)	67 (31-80)
Staging UC (plus accompanying Cis) (n)									
Ta	110	35	-	-	-	-	-	-	-
T1	84 (5 Cis)	17	-	-	-	-	-	-	-
T2	5 (1 Cis)	1	-	-	-	-	-	-	-
T2a/b	26 (1 Cis)/1	8	-	-	-	-	-	-	-
Cis only	6	-	-	-	-	-	-	-	-
Grading UC (n)									
Low grade	173	45	-	-	-	-	-	-	-
High grade	57	14	-	-	-	-	-	-	-
N/A	2	2	-	-	-	-	-	-	-
Recurrent UC (n)									
No	144	36	73	14	9	32	10	18	19
Yes	88	25	2	-	-	1	-	52	10
N/A	-	-	-	-	-	-	1	-	-
Staging PC (n)									
T1c	-	-	25	-	-	-	-	-	-
T2	-	-	1	-	-	-	-	-	-
T2a/c	-	-	1/28	-	-	-	-	-	-
T3a/b	-	-	5/3	-	-	-	-	-	-
N/A	-	-	12	-	-	-	-	-	-
Gleason Score PC (n)									
≤6	-	-	27	-	-	-	-	-	-
7	-	-	35	-	-	-	-	-	-
≥8	-	-	10	-	-	-	-	-	-
N/A			3						
Grading PC (n)									
G1	-	-	27	-	-	-	-	-	-
G2	-	-	27	-	-	-	-	-	-
G3	-	-	8	-	-	-	-	-	-
G4	-	-	2	-	-	-	-	-	-
G5	-	-	8	-	-	-	-	-	-
N/A	-	-	3	-	-	-	-	-	-
Staging RC (n)									
T1a/b	-	-	-	2/10	3/3	-	-	-	-
T2a	-	-	-	1	1	-	-	-	-
T3a	-	-	-	1	2	-	-	-	-
Grading RC (n)									
G1	-	-	-	3	5	-	-	-	-
G2	-	-	-	10	3	-	-	-	-
G3-G4	-	-	-	-	1	-	-	-	-
N/A	-	-	-	1	-	-	-	-	-
Leukocytes in urine (n)									
0	128	10	60	9	5	26	6	32	9
25 Leu/µL	58	23	9	1	-	3	2	14	3
100 Leu/µL	21	9	2	3	3	1	3	6	4
500 Leu/µl	18	18	2	1	1	1	-	14	12
N/A	7	1	2	-	-	2	-	4	1

Table S2. Reduced dataset (excluding specimens >500 leukocytes/µL) utilized to test the performance of the BLSP amplicon in urine. The study population was stratified for a history of urothelial carcinoma, tumour classification and urinary leukocyte counts. Specimens where these data were missing were also excluded. UC=urothelial carcinoma; UCt=urological controls; PCt=population controls; PC=prostate cancer; RC=renal cancer; Cis=carcinoma in situ.

	**UC**		**PC**	**RC**		**PCt**		**UCt**	
	**Male**	**Female**	**Male**	**Male**	**Female**	**Male**	**Female**	**Male**	**Female**
n	207	42	71	13	8	30	11	52	16
Age in years; Median (range)	72 (35-92)	72 (48-89)	68 (48-82)	71 (50-82)	70 (59-82)	71 (48-87)	66 (46-80)	70 (45-86)	66 (31-79)
Staging UC (plus accompanying Cis) (n)									
Ta	100	26	-	-	-	-	-	-	-
T1	78 (4 Cis)	12	-	-	-	-	-	-	-
T2	1 (1 Cis)	-	-	-	-	-	-	-	-
T2a/b	21 (1 Cis)/1	4	-	-	-	-	-	-	-
Cis only	6	-	-	-	-	-	-	-	-
Grading UC (n)									
Low grade	159	34	-	-	-	-	-	-	-
High grade	46	7	-	-	-	-	-	-	-
N/A	2	1	-	-	-	-	-	-	-
Recurrent UC (n)									
No	127	25	69	13	8	29	10	12	10
Yes	80	17	2	-	-	1	-	40	6
N/A	-	-	-	-	-	-	1	-	-
Staging PC (n)									
T1c	-	-	24	-	-	-	-	-	-
T2	-	-	1	-	-	-	-	-	-
T2a/c	-	-	1/27	-	-	-	-	-	-
T3a/b	-	-	4/3	-	-	-	-	-	-
N/A	-	-	11	-	-	-	-	-	-
Gleason Score PC									
≤6	-	-	25	-	-	-	-	-	-
7	-	-	34	-	-	-	-	-	-
≥8	-	-	10	-	-	-	-	-	-
N/A	-	-	2	-	-	-	-	-	-
Grading PC (n)									
G1	-	-	25	-	-	-	-	-	-
G2	-	-	26	-	-	-	-	-	-
G3	-	-	8	-	-	-	-	-	-
G4	-	-	2	-	-	-	-	-	-
G5	-	-	8	-	-	-	-	-	-
N/A	-	-	2	-	-	-	-	-	-
Staging RC									
T1a/b	-	-	-	2/9	3/2	-	-	-	-
T2a	-	-	-	1	1	-	-	-	-
T3a	-	-	-	1	2	-	-	-	-
Grading RC									
G1	-	-	-	3	5	-	-	-	-
G2	-	-	-	9	2	-	-	-	-
G3-G4	-	-	-	-	1	-	-	-	-
N/A	-	-	-	1	-	-	-	-	-
Leukocytes in urine (n)									
0	128	10	60	9	5	26	6	32	9
25 Leu/µL	58	23	9	1	-	3	2	14	3
100 Leu/µL	21	9	2	3	3	1	3	6	4

Table 3. Compilation of significant results from the comparison of urothelial cancer (UC) cell lines with prostate cancer (PC) and renal cancer (RC) cell lines and bladder, prostate and kidney primary cells (in vitro, light green) together with data from previous urine arrays comparing UC patients with population controls (PCt) and urological controls (UCt) (in vivo, light red) [15]. Sites with >20% methylation difference are printed bold.

Locus characteristics				UC vs. PC, RC and primary cells (t-test)			UC vs. PCt (array results)			UC vs. UCt (array results)		
**Array ID**	**UCSC RefGene-Name**	**Chromosome**	**Location**	**DiffBeta**	**P-Value**	**adj. P-Value**	**DiffBeta**	**P-Value**	**adj. P-Value**	**DiffBeta**	**P-Value**	**adj. P-Value**
**cg01742627**		**6**	**28911468**	**0,895979**	**1,27E-08**	**0,000014**	**0,517466**	**0,000082**	**0,009084**	**0,290525**	**0,025502**	**0,153935**
**cg05127899**		**6**	**28911474**	**0,779505**	**7,01E-07**	**0,000351**	**0,467040**	**0,000187**	**0,011318**	**0,329178**	**0,007486**	**0,085506**
**cg11095122**	**CSGALNACT1**	**8**	**19540734**	**-0,516538**	**9,52E-05**	**0,019603**	**-0,326769**	**0,000382**	**0,014600**	**-0,237700**	**0,021454**	**0,140494**
**cg00532451**	**OBSCN**	**1**	**228528913**	**-0,724584**	**8,95E-07**	**0,000418**	**-0,416221**	**0,000001**	**0,005211**	**-0,327530**	**0,000799**	**0,052019**
**cg20775840**	**G3BP1**	**5**	**151150431**	**0,589082**	**3,64E-05**	**0,008497**	**0,219690**	**0,009495**	**0,072115**	**0,271406**	**0,032297**	**0,175020**
**cg04070987**	**NCRNA00162**	**21**	**46424834**	**-0,617919**	**1,97E-05**	**0,005405**	**-0,241029**	**0,006827**	**0,059506**	**-0,339885**	**0,000521**	**0,052019**
**cg13508800**	**COBL**	**7**	**51376950**	**-0,764644**	**1,44E-07**	**0,000084**	**-0,276447**	**0,015712**	**0,097555**	**-0,374799**	**0,000573**	**0,052019**
**cg20657674**		**5**	**180600912**	**0,658108**	**1,90E-04**	**0,034938**	**0,202856**	**0,010011**	**0,074463**	**0,218697**	**0,000063**	**0,052019**
cg01635063	PALM	19	728385	-0,369274	1,43E-04	0,027687	-0,229567	0,002658	0,035436	-0,127094	0,116944	0,357986
cg05839235	NPR3	5	32712237	0,614983	1,46E-05	0,004727	0,328764	0,000046	0,007901	0,163625	0,030391	0,169179
cg07101909		16	3202077	0,906737	6,91E-10	0,000005	0,276452	0,084827	0,281982	0,141601	0,091283	0,312628
cg26885517		16	89314117	0,874773	7,44E-09	0,000013	0,271360	0,073040	0,257009	0,126886	0,055421	0,235870
cg06396762		16	3202609	0,812199	1,41E-08	0,000014	0,264238	0,005852	0,054447	0,141762	0,028191	0,162334
cg25181651	PXDN	2	1748132	0,844807	1,01E-07	0,000071	0,246614	0,028630	0,141759	0,191887	0,116352	0,357065
cg19238520		6	28921359	0,489753	1,55E-05	0,004727	0,213191	0,020879	0,116216	0,071167	0,467791	0,705157
cg23586595	PLAC8	4	84034390	-0,836370	1,18E-08	0,000014	-0,266392	0,040757	0,177384	0,009938	0,941334	0,974858
cg07475178	LDHD	16	75149110	-0,291005	2,59E-04	0,045332	-0,172780	0,091261	0,294979	-0,220721	0,025404	0,153618
cg15788231	COBL	7	51287512	-0,495430	2,01E-05	0,005405	-0,189040	0,025804	0,132810	-0,234354	0,036105	0,185883
cg08534653	PXDN	2	1747700	0,683752	5,46E-07	0,000294	0,157898	0,003755	0,042570	0,180153	0,050901	0,224918
cg06759058		6	28602864	0,483308	2,57E-06	0,001123	0,139213	0,003136	0,038673	0,128393	0,103138	0,334217
cg01252672	NPR3	5	32711881	0,890383	3,68E-08	0,000032	0,191252	0,086138	0,284633	0,174480	0,029670	0,166988
cg26730369	ADRA1A	8	26724001	0,558598	1,46E-04	0,027687	0,176286	0,028753	0,142121	0,158806	0,017898	0,128127
cg03307893	ATP10A	15	26108683	0,404581	3,80E-05	0,008579	0,167347	0,117027	0,343479	0,104094	0,114835	0,354611
cg27380429		10	102498823	0,615784	1,17E-04	0,023389	0,121582	0,063037	0,234034	0,095065	0,016351	0,122458
cg12634446		8	67026411	0,736483	8,29E-08	0,000064	0,100835	0,161040	0,414755	0,176333	0,112222	0,350138
cg10914789	DUSP9	X	152909097	0,514106	6,20E-05	0,013159	0,082472	0,152258	0,401256	0,025223	0,586261	0,784217
cg19913430		8	67025991	0,701773	1,38E-07	0,000084	0,082228	0,480431	0,729111	0,156485	0,063381	0,254734
cg07502439	SALL1	16	51185461	0,729335	2,33E-05	0,006032	0,077703	0,216890	0,489609	0,119830	0,032470	0,175538
cg25571269	MIR193A	17	29886758	0,593454	1,17E-05	0,004198	0,071827	0,205170	0,475114	0,095216	0,078476	0,287336
cg01583134		8	67026111	0,514104	4,85E-06	0,001884	0,032554	0,548020	0,773997	0,115962	0,134315	0,385721
cg23912878	MBIP	14	36789691	0,337505	2,47E-04	0,044388	0,021346	0,086518	0,285447	-0,023651	0,226450	0,502113
cg22297146	MYEF2	15	48470516	0,424885	3,02E-06	0,001244	0,014628	0,556435	0,779424	0,091719	0,230536	0,506359
cg27057210	MBIP	14	36789810	0,299665	1,55E-05	0,004727	0,003038	0,839107	0,932811	0,009118	0,413871	0,666262
cg21443584	MBIP	14	36789985	0,460295	4,03E-05	0,008821	0,002198	0,921396	0,968543	0,002888	0,940670	0,974642
cg02345266	NKD2	5	1008964	0,295000	2,52E-05	0,006310	-0,000248	0,950240	0,980394	0,010122	0,337012	0,605373
cg26054540	NKD2	5	1009204	0,323628	1,84E-05	0,005374	-0,001366	0,884604	0,952760	0,003821	0,712904	0,859342
cg25222014	C10orf88	10	124714072	0,884367	6,84E-09	0,000013	-0,018394	0,039922	0,175070	0,004815	0,412452	0,665219
cg26956371	C10orf88	10	124713989	0,309422	2,89E-05	0,006977	-0,032747	0,078476	0,268798	0,028936	0,182670	0,452231
cg15777964	SIK1	21	44847606	0,918199	4,64E-09	0,000013	-0,034258	0,898698	0,958975	0,124577	0,070663	0,270942
cg19697575	44	2	172374119	-0,610542	1,20E-05	0,004198	-0,198207	0,082528	0,277251	-0,022845	0,839535	0,927072

Table S4. p-values and median differences (Mann Whitney test) for all measurable CpGs of the bladder-specific (BLSP) amplicon when comparing the UC group with all other groups *in the large urine dataset* from Table S1.

		Comparison	CpG 5.6	CpG 7	CpG 8	CpG12
**Men**	p-values	UC vs. PC	<0.0001	<0.0001	<0.0001	<0.0001
		UC vs. RC	<0.0001	<0.0001	<0.0003	<0.0003
		UC vs. PCt	<0.0001	<0.0001	<0.0001	<0.0001
		UC vs. UCt	<0.0001	<0.0001	<0.0001	<0.0001
		UC vs. ∑other groups	<0.0001	<0.0001	<0.0001	<0.0001
	median differences [%]	UC vs. PC	19.0	22.0	24.0	16.0
		UC vs. RC	18.5	22.0	24.0	15.5
		UC vs. PCt	19.0	21.0	23.0	17.0
		UC vs. UCt	22.0	24.0	27.5	17.0
		UC vs. ∑other groups	19.0	22.0	25.0	17.0
**Women**	p-values	UC vs. RC	0.1982	0.2213	0.0003	0.0003
		UC vs. PCt	0.0617	0.2389	0.4790	0.0189
		UC vs. UCt	0.006	0.0025	0.0051	<0.0001
		UC vs. ∑other groups	0.0004	0.0028	0.0181	<0.0001
	median differences [%]	UC vs. RC	2.0	2.0	24.0	15.5
		UC vs. PCt	3.0	2.0	2.0	4.0
		UC vs. UCt	4.0	4.0	5.0	5.0
		UC vs. ∑other groups	3.0	4.0	4.0	4.0

Table S5. p-values and median differences (Mann Whitney test) for all measurable CpGs of the bladder-specific (BLSP) amplicon *in the reduced dataset* from Table S2.

		Comparison	CpG 5.6	CpG 7	CpG 8	CpG12
**Men**	p-values	UC vs. PC	<0.0001	<0.0001	<0.0001	<0.0001
		UC vs. RC	<0.0001	<0.0001	<0.0001	<0.0001
		UC vs. PCt	<0.0001	<0.0001	<0.0001	<0.0001
		UC vs. UCt	<0.0001	<0.0001	<0.0001	<0.0001
		UC vs. ∑other groups	<0.0001	<0.0001	<0.0001	<0.0001
	Median differences [%]	UC vs. PC	25.0	25.0	29.0	20.0
		UC vs. RC	23.0	26.0	27.0	19.0
		UC vs. PCt	24.5	25.0	28.0	21.0
		UC vs. UCt	27.0	28.0	31.5	21.0
		UC vs. ∑other groups	25.0	26.0	29.0	21.0
**Women**	p-values	UC vs. RC	0.2917	0.3367	0.7208	0.2403
		UC vs. PCt	0.0399	0.1538	0.3363	0.0105
		UC vs. UCt	0.0083	0.0095	0.0377	0.0500
		UC vs. ∑other groups	0.0033	0.0100	0.0693	<0.0001
	median differences [%]	UC vs. RC	1.5	1.5	0.5	1.5
		UC vs. PCt	3.0	2.0	1.5	4.0
		UC vs. UCt	3.0	5.0	3.5	5.0
		UC vs. ∑other groups	3.0	3.0	1.5	4.0

Table S6. p-values (Anova, Kruskal-Vallis-Test) for all unambiguous and robustly measurable CpGs of BLSP *in the reduced urine dataset* from Table S2.

		Comparison	CpG 5.6	CpG 7	CpG 8	CpG12
**Men**	p-values	PC vs. RC vs. UCt vs. PCt	0.4126	0.8783	0.8652	0.0864
		UC vs. ∑other groups	<0.0001	<0.0001	<0.0001	<0.0001
**Women**	p-values	RC vs. UCt vs. PCt	0.3884	0.2467	0.1548	0.0283
		UC vs. ∑other groups	0.0224	0.0424	0.1331	0.0002

Table S7. DNA-methylation value cutoffs for the identification of UCa among control individuals, prostate and renal cancer patients (UCt, PCt, PCa, RCa) *in the reduced urine dataset* from Table S2. The cutoffs were calculated for a minimum specificity of 95%.

		Comparison	CpG 5.6	CpG 7	CpG 8	CpG12
**Men**	Cutoff optimised for specificity	% specificity	96.14	95.17	95.65	95.17
		% sensitivity	29.52	32.73	21.08	18.07
		Cutoff	0.0450	0.0650	0.0950	0.0750
**Women**	Cutoff optimised for specificity	% specificity	95.24	95.24	95.24	100
		% sensitivity	8.57	8.57	0.00	8.57
		Cutoff	0.0250	0.0250	0.0500	0.0250

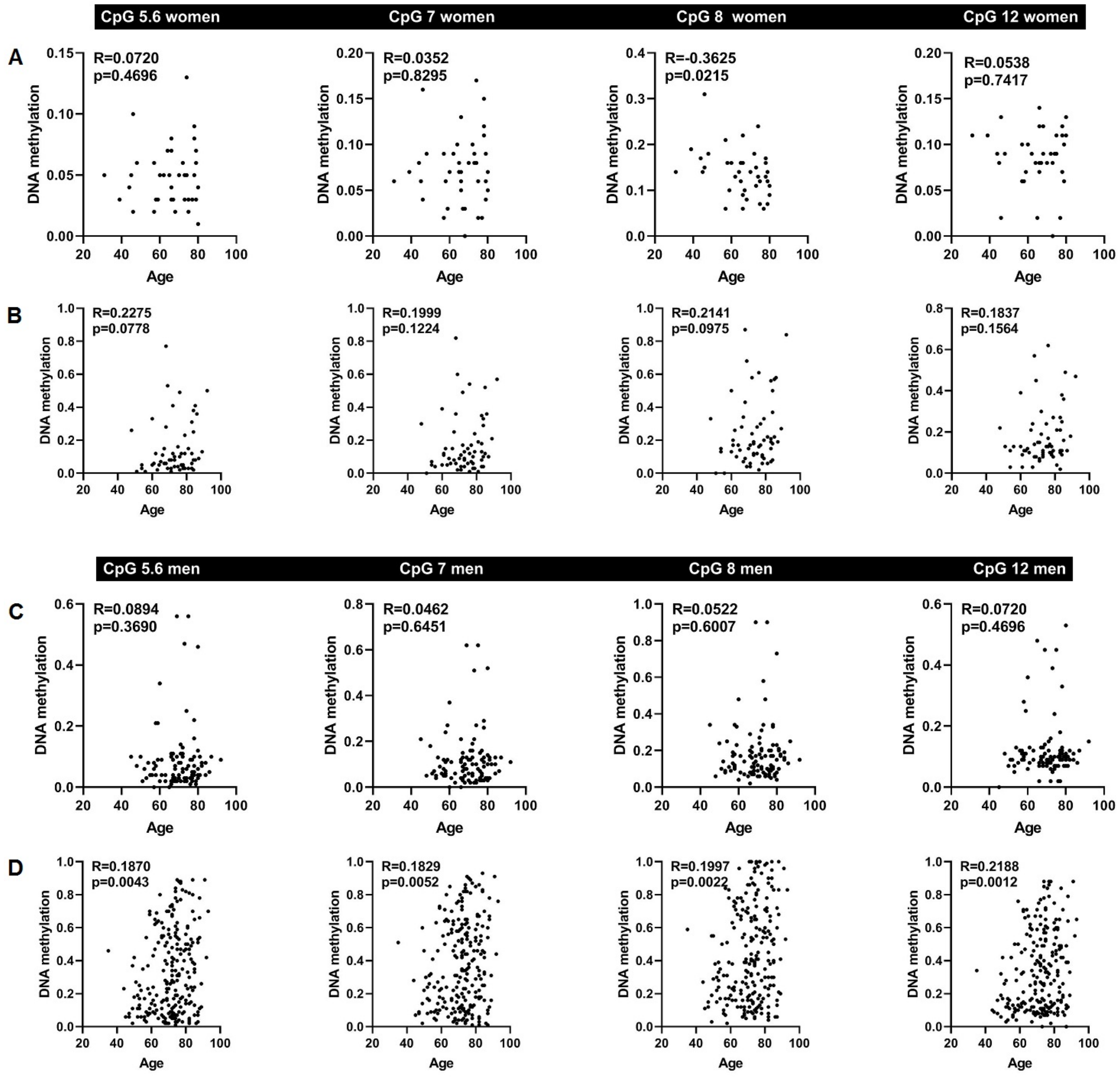

Figure S1. Urinary DNA-methylation values and association with age in female controls (**A**) and UC patients (**B**), and male controls (**C**) and UC patients (**D**).

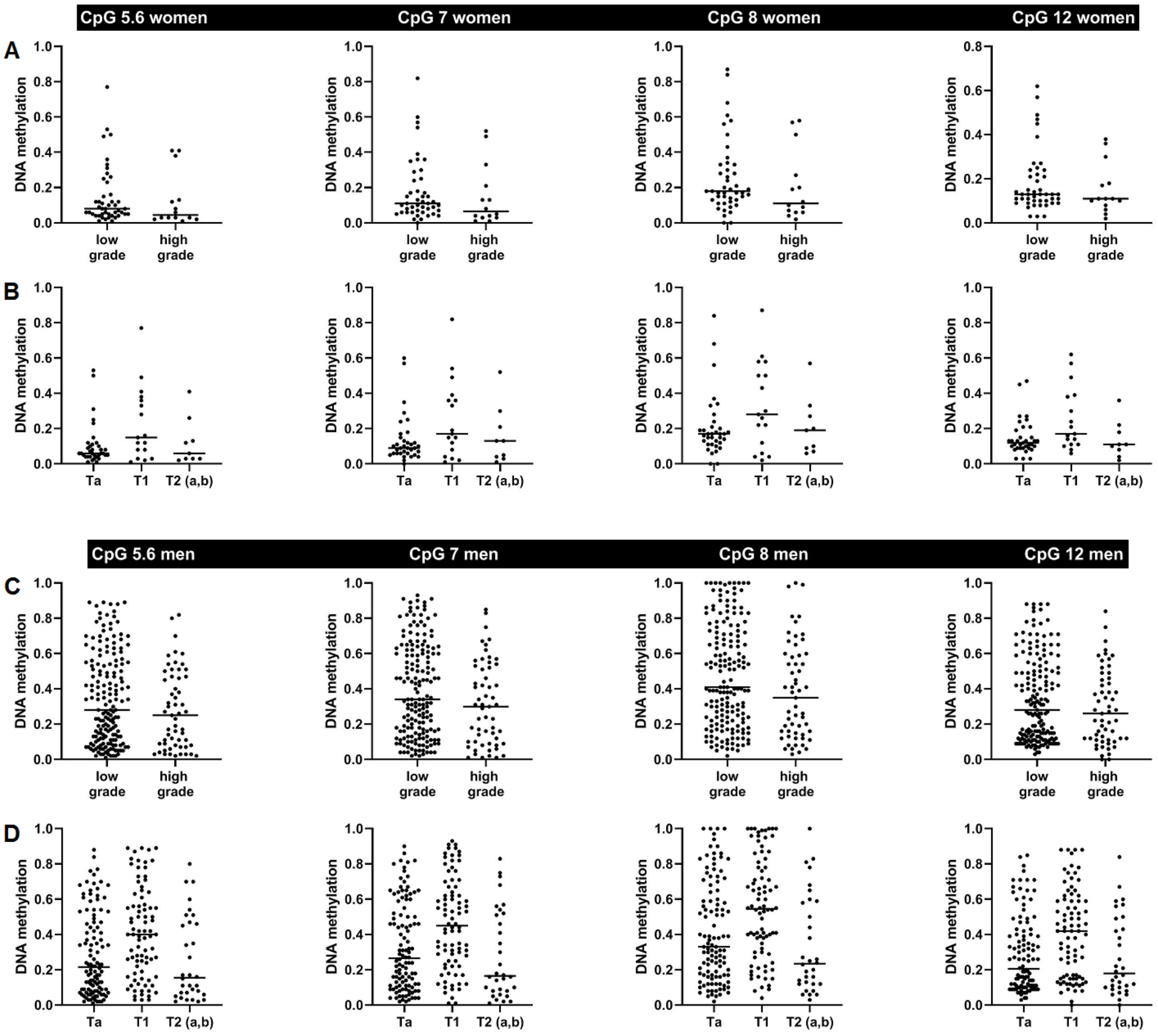

Figure S2. Urinary DNA-methylation values and relationship with grade (**A**) and stage (**B**) in female UC patients, and grade (**C**) and stage (**D**) in male UC patients. Horizontal bars represent the median DNA methylation value.

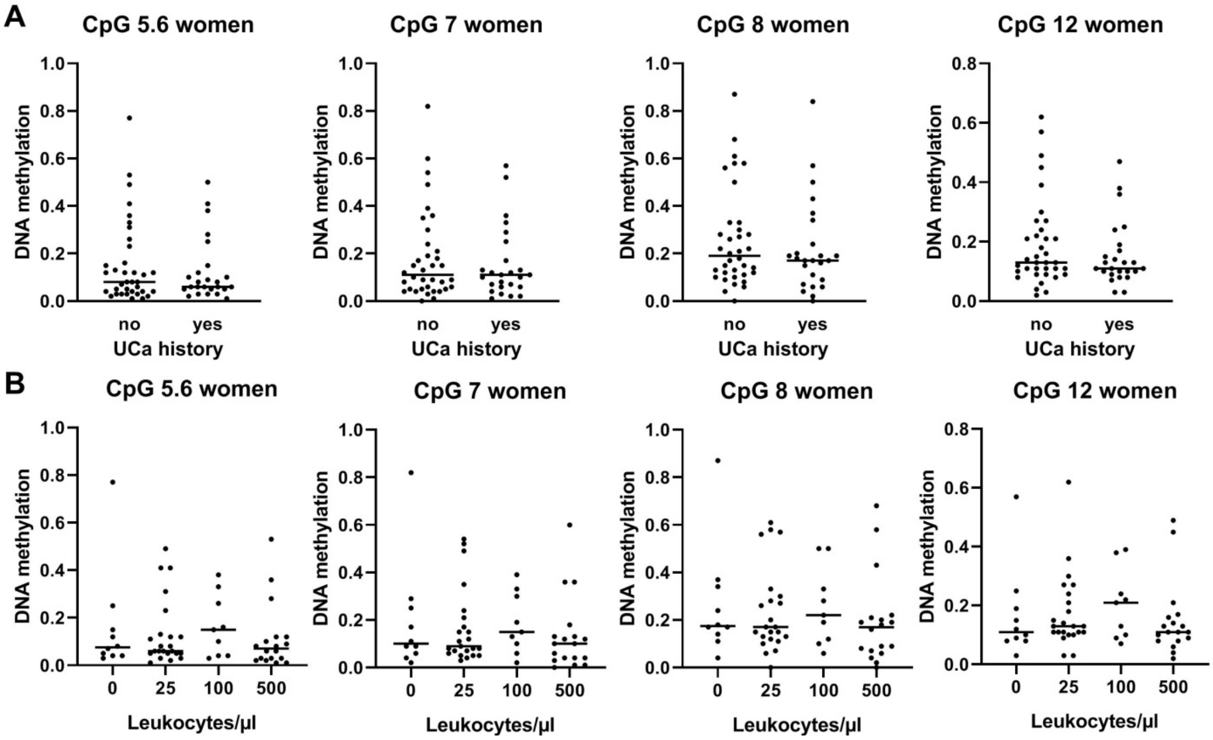

Figure S3. Urinary DNA-methylation values of female UC patients; the data are stratified for UC history (**A**) and urinary leukocyte counts (**B)**. Horizontal bars represent the median DNA methylation value.

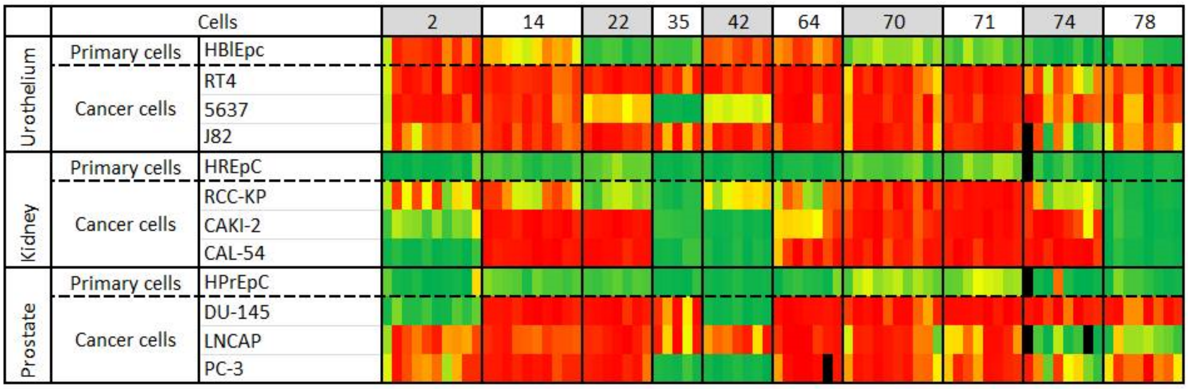

Figure S4. CpG-resolved DNA-methylation for the amplicons presented in our previous study [15] when assessed in the cancer cell lines and primary cells utilized in the present study. Increasing DNA methylation values are encoded by a color gradient from 0% methylated (green) to 100% methylated (red). Black = no value obtained.

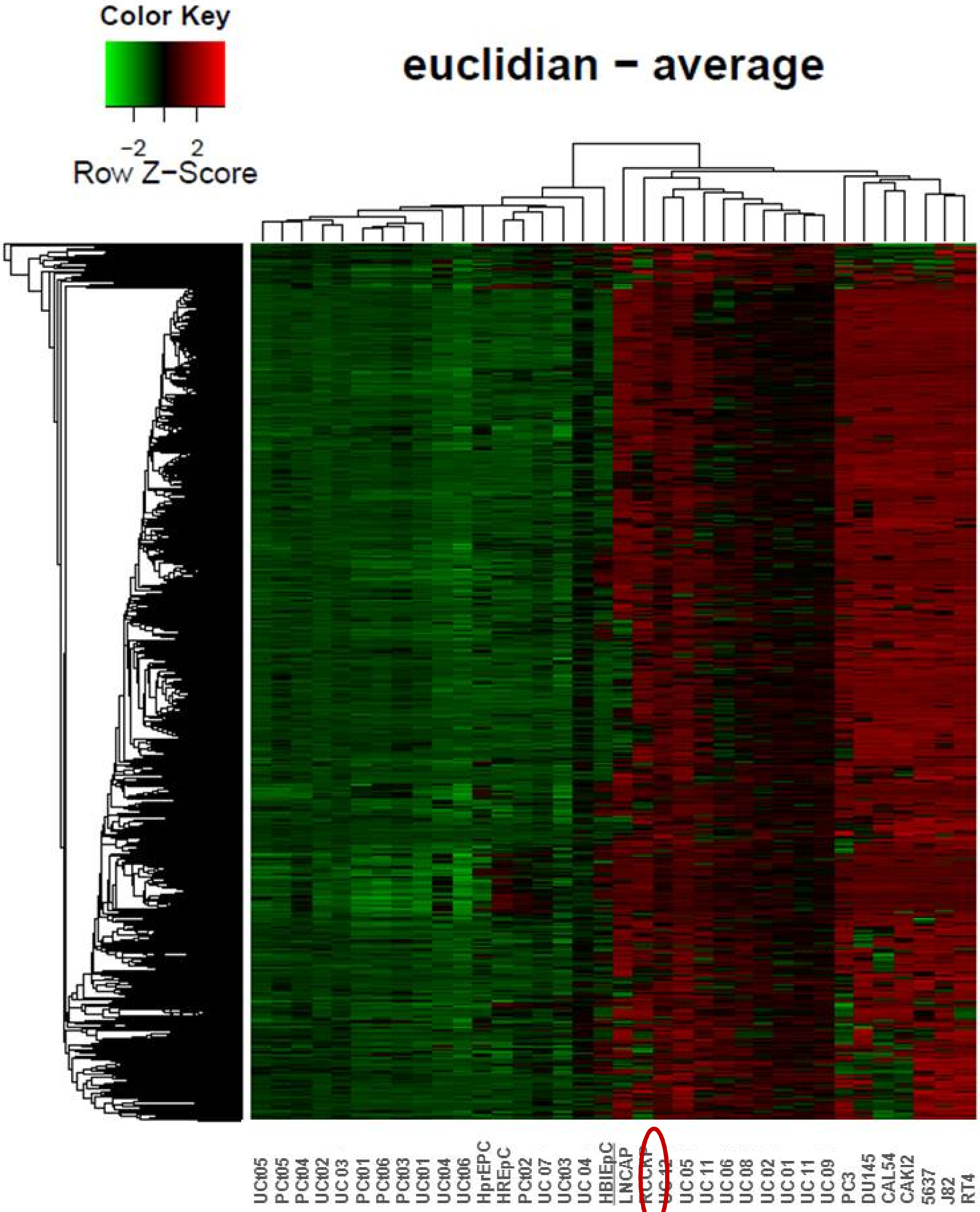

Figure S5. Integrated heatmap of the infinium 450K results for all primary cells and cancerous cell lines in the present study and those from urine samples previously analyzed [15]. The results show that HBIEpC (primary urothelial cells, red circle) share similarities with both, UC patients and cancer cell lines (mostly to the right of HBIEpC) and RC/PC primary cells and PCt/UCt controls (mostly to the left of HBIEpC).

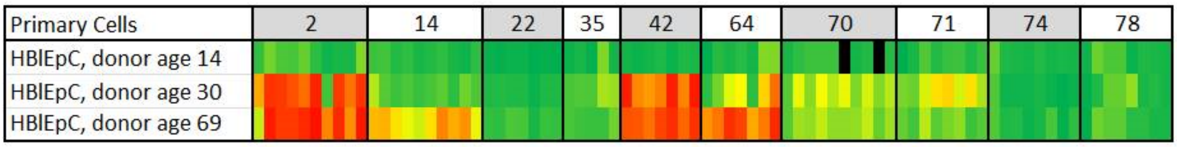

Figure S6. CpG-resolved DNA-methylation for the amplicons presented in our previous study [15] when assessed in primary urothelial cells obtained from donors of different ages. Increasing DNA methylation values are encoded by a color gradient from 0% methylated (green) to 100% methylated (red). Black = no value obtained.
